# Pragmatic Frames for Teaching and Learning in Human–Robot Interaction: Review and Challenges

**DOI:** 10.3389/fnbot.2016.00010

**Published:** 2016-10-03

**Authors:** Anna-Lisa Vollmer, Britta Wrede, Katharina J. Rohlfing, Pierre-Yves Oudeyer

**Affiliations:** ^1^FLOWERS Team, Inria, France; ENSTA ParisTech, France; ^2^Applied Informatics Group, CITEC, Bielefeld University, Bielefeld, Germany; ^3^Psycholinguistics Group, Paderborn University, Paderborn, Germany

**Keywords:** robot learning, robot teaching, human–robot interaction, pragmatic frames, social learning, language learning, action learning, cognitive developmental robotics

## Abstract

One of the big challenges in robotics today is to learn from human users that are inexperienced in interacting with robots but yet are often used to teach skills flexibly to other humans and to children in particular. A potential route toward natural and efficient learning and teaching in Human-Robot Interaction (HRI) is to leverage the social competences of humans and the underlying interactional mechanisms. In this perspective, this article discusses the importance of pragmatic frames as flexible interaction protocols that provide important contextual cues to enable learners to infer new action or language skills and teachers to convey these cues. After defining and discussing the concept of pragmatic frames, grounded in decades of research in developmental psychology, we study a selection of HRI work in the literature which has focused on learning–teaching interaction and analyze the interactional and learning mechanisms that were used in the light of pragmatic frames. This allows us to show that many of the works have already used in practice, but not always explicitly, basic elements of the pragmatic frames machinery. However, we also show that pragmatic frames have so far been used in a very restricted way as compared to how they are used in human–human interaction and argue that this has been an obstacle preventing robust natural multi-task learning and teaching in HRI. In particular, we explain that two central features of human pragmatic frames, mostly absent of existing HRI studies, are that (1) social peers use rich repertoires of frames, potentially combined together, to convey and infer multiple kinds of cues; (2) new frames can be learnt continually, building on existing ones, and guiding the interaction toward higher levels of complexity and expressivity. To conclude, we give an outlook on the future research direction describing the relevant key challenges that need to be solved for leveraging pragmatic frames for robot learning and teaching.

## 1 Introduction

Robots have long been predicted to become everyday companions capable to help and assist us in our daily tasks. A major challenge to achieve this vision is to enable robots to learn new tasks through natural social interaction with (non-expert) humans. So far, however, robots are designed for specific purposes and can therefore barely handle the diversity of learning and teaching cues used by humans to acquire sensorimotor and linguistic skills (cf., Amershi et al. (2014) for an overview). Unsolved issues are linked to the necessity to know which information to use for learning, how to behave naturally in interaction, and how to create common ground^1^ in communication.

Some studies on *human development* recognize social interaction as facilitating learning processes by providing a stable structure (see a summary in Rohlfing et al. (2016)). This stable structure is described by the concept of *pragmatic frames*, which has been introduced by Bruner (1983) and recently been re-introduced by Rohlfing et al. (2016). Accordingly, pragmatic frames are recurrent patterns of interaction that are constituted by a set of sequentially organized actions (including verbal, non-verbal, and multimodal behavior which can also occur in parallel) and support children in language acquisition (cf., Box 1 on conceptual definitions). Leveraging pragmatic frames for robotics might have the potential to overcome the open challenges in robot learning of sensorimotor and linguistic skills.

Box 1 **Conceptual definitions.**

This box explains the definition of relevant terms frequently used throughout this paper. It is useful to understand and clarify better the concept of pragmatic frames (which can appear rather vague) as the basis of our investigations. The definitions are adapted from Rohlfing et al. (2016).

**Learning/teaching pragmatic frame**

A pattern in verbal and non-verbal behavior involving goal-oriented actions coordinated with the interaction partner that emerges over recurrent interactions.

Recall the previous example of Bruner’s book-reading frame (Bruner, 1983), which consists of (1) the parent directing the child’s attention, (2) the child paying attention, (3) the parent asking the child for a label, (4) the child answering according to her capabilities, and (5) the parent providing feedback and (6) the correct label (see Table S5 in Supplementary Material).

A learning/teaching frame like the above involves a teacher explicitly teaching a certain content (e.g., a word or an action) to the learner.

In contrast, in current human–robot interactions for teaching robot sensorimotor skills, pragmatic frames are predefined by the designer or developer including a lot of implicit prior knowledge about the action and often involve the developer to provide relevant structuring information for the learning robot system. As an example, consider the experiment of Calinon et al. (2010) where a robot learned through kinesthetic teaching to feed a doll with a spoon. In that frame, (1) the robot gazed straight ahead with a spoon in its hand and was prepared by preprograming to focus on the trajectory of the arm movement and the objects doll and plate: the doll was rigidly attached to the robot, adding a link to the robot’s kinematic chain, and it received data from an external vision system tracking the marker attached to the plate; (2) the experimenter/developer provided the signal for the beginning of the teaching, i.e., recording the data; (3) the tutor provided the arm movements through kinesthetic teaching; (4) the experimenter ended the recording and started the learning process; (5) the robot used the visual input and proprioception to derive the position of the landmarks (doll’s mouth, plate) to normalize the trajectories with respect to these landmarks; (6) the robot used the normalized trajectories to update the parameters of a HMM/GMM model; (7) the experimenter started the action execution behavior; and (8) the robot executed the action based on the updated model parameters.

**Syntax of a teaching/learning pragmatic frame**

We call the sequence of verbal and non-verbal actions that characterize the appearance of a pragmatic frame the syntax of the pragmatic frame, while Bruner refers to it with the term *structure* of a format (Bruner, 1983). We define the syntax as the observable sequence of behaviors constituting the pragmatic frame. The syntax contains among others the adequate sensory means, possible orders of behavioral units, and information about actors with which the pragmatic frame is realized. The syntax of a frame is highly conventionalized and can thus vary from person to person and from culture to culture. The form of the parts that constitute the syntax is variable with respect to the utterances and tokens used. Coming back to the book-reading frame described above, possible tokens include: X (=label), Its an X, Thats an X, There is an X, etc. (cf., Bruner (1983), p. 79); intonation, prosody, pause lengths, etc. Young infants, however, are presented with a stable caregiver’s behavior on which they rely. In teaching/learning frames, the syntax also specifies the slot for the learning content (i.e., where it is in the sequence) and the type of content (e.g., a noun is learned or a color is learned). This information about slot and type of learning content links the syntax to the meaning of a pragmatic frame.

The syntax of the pragmatic frame in the teaching example by Calinon et al. (2010) consists of the observable sequence of behaviors, i.e., the researcher activating the start/end button, the tutor providing the action demonstration in the correct way (i.e., only providing correct demonstrations and refraining from providing negative examples), etc. The slot in this example pertains to the movement trajectory relative to the positions of the landmarks.

**Meaning of a teaching/learning pragmatic frame**

We call the set of effects that a frame has on memory processes^1^ (i.e., the cognitive operations involved in the frame) when learning new skills (e.g., acquiring new words or actions) the meaning of a pragmatic frame. Whereas, of course, memory effects are present for both interaction partners, we focus on the learner side. These cognitive operations are recruited from memory to allow own behaviors to be triggered by conventionalized signals (i.e., individual elements of behavioral patterns that are known to the learner from previous interactions, already acquired pragmatic frames, or constituents of such) and to process the learning content of the pragmatic frame. The meaning in our book-reading frame example could be composed of image segmentation, classification, association of the label to the internal representation, etc. The meaning can be basic and automated in terms of being composed of reactive behaviors but bears some dispositions that are co-constructed with the partner: For example, when the tutor points to an object, the learner not only follows the gesture but also expects a referent (Gliga and Csibra, 2009). The meaning thus creates expectation/anticipation toward the learning content at the cognitive level.

In the robot teaching example (Calinon et al., 2010), the cognitive processes consist of all the perception algorithms involved in the scenario (i.e., the visual tracking of the plate marker, the proprioception of the arm joints, etc.) as well as the transfer of the relevant data to the relevant part of the learning algorithm, i.e., the arm movement data in the chosen learning space dimension to update the HMM parameters and the landmark positions and arm movement data to update the GMR model that represents the movement constraints and allows for two landmarks.

**Learning content of a teaching/learning pragmatic frame**

Whereas not every pragmatic frame has a learning content (e.g., a greeting frame, peek-a-boo), in the tutoring interaction context, the learning content is the information that should be transferred from teacher to learner. It is what the teacher wants to teach the learner, as for example, the labels for objects in Bruner’s book-reading frame. The learning content in the robot teaching example in Calinon et al. (2010) should consist of the information relevant to generalizing the demonstrated action (i.e., what is important about the action), however, it basically consists of the form of the trajectories from home position to landmark 1 and from landmark 1 to landmark 2. In this case, what is learned is how the plate landmark can be reached from different starting positions and how the mouth landmark can be reached from different plate landmarks. It does not contain the implicitly given information about the positions of the landmarks and the information about their sequence, as well as the structure of the action (i.e., path-oriented action where constraints of landmarks need to be met).

**Slot in a teaching/learning pragmatic frame**

The slot of a pragmatic frame is the place in the interactional sequence holding the variable learning content, which the learner can pick up. The slot is embedded in a familiar fixed sequence constituting the frame. In the example of Bruner’s book-reading frame, which we revisited in the definition of a learning/teaching pragmatic frame, the slot is step (6) in the sequence of behaviors in which the parent utters the correct label of the relevant image (i.e., the learning content). The slot is specified by the syntax of the frame, and thus, when a pragmatic frame is learned, the slot is learned together with the syntax of the frame. In the robot teaching example (Calinon et al., 2010), the slot is step (3) of the pragmatic frame described above in the definition of a learning/teaching pragmatic frame, in which the user provides the kinesthetic demonstrations of arm movements.

**Learning scope of a teaching/learning pragmatic frame**

Once a frame is established, learning sensorimotor and linguistic skills (e.g., an action or word) within this frame does not extend to making sense of a whole sequence of observable behavior. Instead, the structure of the pragmatic frame constrains the learning hypotheses such that learning is limited to (1) the relation of specific observable features within the slot (e.g., the auditory information making up the label in the book-reading frame) with specific features of the underlying concept (e.g., the visual appearance of a segmented object in the area of joint attention, such as the area of the book page the mother is pointing to in the book-reading frame) or (2) learning the concept that underlies the cognitive operations within a specific frame (e.g., learning to identify features regarding the shape of an object vs. features capturing attributes such as color). In the robot teaching example, the learning scope relates to the generalization of a movement from one landmark to another for different positions.

**Format**

Bruner’s term for pragmatic frames he observed in adult–child interactions the “principle vehicle” of the “Language Acquisition Support System” framing the interaction such that it helps the child to learn language. He states that “A format is a standardized, initially microcosmic interaction pattern between an adult and an infant that contains demarcated roles” and over time becomes a familiar routine (Bruner (1983), p. 120 f.).

**Language game**

Pragmatic frames bear resemblance of what Wittgenstein (1953) calls “language games [Sprachspiele].” He defines them as protocols or scripts in which action and language are interwoven to result in a behavioral disposition in the interlocutor. The notion of language games was introduced to the field of robotics by Steels (2001) for approaches to language evolution. Pre-programed interaction protocols were specifically designed to allow robots to learn language. According to Steels, a language game is a “routinized sequence of interactions between two agents involving a shared situation in the world” (Steels and Kaplan (2002), p. 9), promoting grounding by creating a context that limits the possible meanings of words (Steels, 2001).

^1^We use the terms “cognitive operations” and “memory processes” interchangeably.

We detail an example of a pragmatic frame in the following. Bruner (1983) studied a book-reading frame, where a mother reads a picture book to her son in a natural setting. The parent first directs the child’s attention to one of the images in the book by means of pointing and saying “Look!,” for example. Then, she asks what the child sees on the image with a query like “What’s that?.” The child then is given the opportunity to respond, but irrespective of the performance, the mother will give feedback (positive feedback most of the time) by saying, for example, “Yes” and gives the child the learning input “It’s a *pineapple*.” This pragmatic frame is about explicit teaching. The mother explicitly teaches her son words for the things depicted in the book. The word “pineapple” in the previous example is the label the child is supposed to learn and thus represents the learning content (i.e., the input) of the pragmatic frame. There are many dimensions to pragmatic frames, and we will not regard all of them but focus on their confined learning scope or more precisely on the extent to which the structure of pragmatic frames provides cues to learning new words and actions. Frames such as the above thus are the kind of teaching/learning frames for acquiring sensorimotor and linguistic skills, which form the ground for the starting point of our analysis. Other early frames Bruner describes, which are not teaching/learning frames, are frames in form of well-known games such as, for example, peekaboo or the knee-ride games “This is the way the ladies ride” and “Ride a cock horse.” Although children clearly can also learn a lot in these pragmatic frames, they do not involve a specific learning content such as the label in the book-reading frame and involve different cognitive functions.

Pragmatic frames contain roles with tasks that are distributed among the interaction partners. For example, for teaching/learning, an action such as a movement with an object, the teacher’s role implies to first get the learner’s attention and to make an object visible (by, e.g., pointing to it or lifting it up); the learner’s role, in turn, implies to follow the pointing and to perceive the highlighted object and then to follow its movement. The tasks corresponding to a role may indicate the target, obstacle, tool, pre-condition, post-condition, etc., of the action. Pragmatic frames provide a learning environment for the child that is familiar and stable. They tend to be instantiated by recurrent presentations bearing hardly any variability and can therefore function as guides for assessing and identifying what the relevant information for learning is. When a child is presented with a familiar frame, the processing is facilitated in the sense that in this familiar stable interaction pattern, the child can easily participate by predicting the next step of interaction and fulfilling his or her role, pick up the information that he or she is supposed to learn (i.e., the learning content), and understand what this information means. For the pragmatic frame of picture book reading, this information is the only variable part of the frame: what is depicted in the image pointed to and the corresponding label. Crucially, even though some aspects of pragmatic frames are certainly culturally motivated (Nelson, 2009), in general, they are emergent, such that the child negotiates and learns new pragmatic frames, and is able to adapt to new forms tailored to her learning progress in repeated interaction with the caregiver.

In the light of the findings presented by Rohlfing et al. (2016), we propose pragmatic frames to be an efficient tool for robot learning language and action from human tutors. Pragmatic frames allow teachers and learners to converge on rich contextual information about the learning content such that the structure of the interaction is actively leveraged for learning sensorimotor and linguistic skills (e.g., conveying what type of information is to be learned and what this information is), allowing efficient and cumulative learning in the long term. The ground for the following review is the hypothesis that a robot learner capable of handling multiple pragmatic frames and even learn and negotiate new ones allows for more flexible and natural interaction, and thus is more easily usable by non-expert users. In this work, we will use the concept of pragmatic frames as a lens to identify and critically analyze the weaknesses of current approaches in robot learning from a human teacher (in comparison with natural human–human interactions). In addition to our analysis, we will outline challenges for future research on creating flexible and adaptive interaction systems allowing rich learning and teaching with pragmatic frames in human-robot interaction (HRI).

## 2 Review of Teaching/Learning Frames Used in the Robot Learning in HRI Literature

The following analyses provide a critical review on pragmatic frames present in the current literature on robot learning in interaction with a human teacher, their use, and the consequences as well as drawbacks tied to the common practice. The review does not aim to be a comprehensive one but tries to cover a variety of approaches. We are aware that our review comprises approaches with a different focus (for example, providing a learning algorithm which solves a certain problem) and compares the approaches nonetheless aiming to criticize a general issue.

As stated above, pragmatic frames consist of syntax and meaning. Much of the syntax actually describes the interaction between the tutor and the learner, i.e., how they coordinate the information exchange. In contrast, the meaning refers to the underlying cognitive processes which, in robotics, are modeled by machine learning approaches. Therefore, to identify the relevant causes for the poorness of pragmatic variation in learning approaches, we need to look (1) at the interactional characteristics of such approaches as well as (2) at the underlying learning algorithms. These two aspects open a complex search space. To structure this search space and to cover a diversity of works, we set up a taxonomy based on the categories developed by two reviews of learning approaches, one from an interactional perspective (Thomaz and Breazeal, 2006a) and one from an algorithmic perspective (Cuayáhuitl, 2015). Based on this taxonomy, we selected 15 papers from the robotics literature focusing on scenarios in which the system is learning from a human teacher who teaches the robot new actions or words. We detail the development of this taxonomy and the selection of the papers in the following Method section.

As we focus only on pragmatic frames for explicit teaching of sensory motor skills and linguistic labels, we restrict our overview to machine learning approaches, targeting sensorimotor skills and linguistic labels. As, certainly, it is possible to learn other things such as social cues in pragmatic frames, there exist other very important works which we do not analyze (e.g., Boucenna et al. (2014a,b), Andry et al. (2001), and Nagai et al. (2003)).

The following questions were central to our analyses: What is the structure of interaction? What information is passed? What are the consequences for the learning algorithms?

### 2.1 Method

We began our review leveraging the categories established in two relevant works. Thomaz and Breazeal (2006a) categorized machine learning approaches from a human–robot interaction perspective. The dimensions they propose include *implicit vs. explicit training* (Is the system passively observing the performance of a human or is a human teacher teaching the robot?), *human vs. machine leading the interaction* (here, the machine leads in approaches of active learning or when employing queries), and the dimension of *human guidance vs. exploration* (human guidance includes learning by demonstration approaches). Cuayáhuitl (2015) groups machine learning frameworks into four categories based on their algorithmic nature: *supervised learning, reinforcement learning, unsupervised learning*, and *active learning*. Our choice of papers about teaching of sensorimotor skills and labels in interaction represents these categories in a balanced manner. As the above categories suggest, in this analysis, we focused not only on the technical side but also on the surface structure of the human–robot interaction. This surface structure represents the syntax of the pragmatic frames used in the paper and will be explicitly described and presented in tables. The meaning of the pragmatic frames is given by the processing of given information and the storage of the learning content. It will be treated by analyzing the usage of information elements in the pragmatic frame (see below) and with the description of the learning algorithm and the processing of the learning input.

This review is not only cataloging information from the papers but presents new insights, as in the vast majority of works, the pragmatic frame is not conceptualized or made explicit.

In the first step, for each work, we determined the focus of the approach and what is being learned. We then related it to the proposed learning algorithm.

In the second step, we analyzed the works according to the observable interactional sequence they presuppose and created a table for each approach (Tables 1–4). For this, we analyzed the kinds of information passed between interactants (robot, user, and experimenter) by identifying common classes of pieces of information (e.g., signals for the start and end of an interaction or the learning input, prompts to perform the learned task, feedback, etc.). We will call these classes *information types*, which do not hold any information on the means or token with which their elements are conveyed (i.e., their *form*) but comprise in part syntax and meaning. In Table 5, we provide a complete list of the information types that we identified in our analysis.

**Table 1:** **Basic common pragmatic frames for the category: passive learning**.

Experimenter/programmer actions	Teaching user actions	Robot learner actions	Robot learning
**Frame 1**			
1. Start	2. Input		Learning
3. End			
**Frame 2**			
1. Start			
		2. Perform	
3. End			

**Table 2:** **Basic common pragmatic frames for the category: exploration learning with initial user demonstration**.

Experimenter/programmer actions	Teaching user actions	Robot learner actions	Robot learning
**Frame 1**			
1. Start	2. Input	3. Act	Learning
4. End			
**Frame 2**			
1. Start			
		2. Perform	
3. End			

*Step (3) and learning repeat*.

**Table 3:** **Basic common pragmatic frames for the category: exploration learning with user refinement**.

Experimenter/programmer actions	Teaching user actions	Robot learner actions	Robot learning
**Frame 1**			
1. Start		2. Act	
	3. Feedback		Learning
4. End			
**Frame 2**			
1. Start			
		2. Perform	
3. End			

*Steps (2) and (3) and learning repeat*.

**Table 4:** **Basic common pragmatic frames for the category: active learning**.

Experimenter/programmer actions	Teaching user actions	Robot learner actions	Robot learning
**Frame 1**			
1. Start		2. Input query	
	3. Input		Learning
4. End			
**Frame 2**			
1. Start			
		2. Perform	
3. End			

*Steps (2) and (3) and learning repeat*.

**Table 5:** **The different information types found in the analyzed literature**.

Information types from human user (or experimenter/developer)	Information types from robot
Start interaction	Confirm
End interaction	Error
Advance sequence	
Direct attention	Attention
Start input	Act
End input	Execute commands
Input	
Correct input	
Input submit	
Prompt input query	Input query
Answer input query	
Prompt input performance	Perform input
Prompt performance	Perform
Prompt feedback	Prompt feedback
Feedback binary	Feedback binary
Feedback correction	Feedback correction

Our interest lay on the following three properties of given information: (1) form (i.e., the surface form of the piece of information; e.g., verbal command, gesture), (2) usage (i.e., how the information is used; e.g., to advance the sequence, for learning, for transparency (cf., Thomaz and Breazeal (2006b))), and (3) flexibility (i.e., the degree of flexibility of the passed information). The usage of the information relates to the meaning of the pragmatic frame, though it does not describe how the information is processed. Regarding the flexibility, information

- can be optional, meaning it can be omitted;
- can be variable with respect to timing, i.e., when in the sequence, it is passed or in which pace, including the number of times a certain subsequence is repeated;
- can have a variable surface form, including, for example, the choice of object;
- can be passed simultaneously in a freer back-and-forth; and
- might not be specified in the sequence beforehand.

In the tabular representation of pragmatic frames, we filter out many of the above properties, such as the usage, and focus on the information types and their flexibility (stated in bold in the tables) in order to reach a common informational representation of the structure of the pragmatic frames. Information types do not only concern the syntax of a pragmatic frame but also bridge the syntax and meaning, such that the structure we depict in the tables represents the surface in syntax and meaning of the pragmatic frame, including when the learning mechanisms come into play.

Third, we placed a special focus on the identification of the implicit knowledge the programmer and experimenter give to the robot to make sense of the learning data. This information might be hard-coded into the system or conveyed as a predefined signal inside the behavior sequence of the pragmatic frame.

For the analysis, we will thus describe the individual works using the following keys of analysis which have been detailed above:

- *Focus*: the focus of the work describes what the paper is concerned with, briefly summarizing its contents.
- *HRI category*: the paper will be classified along the HRI dimensions established by Thomaz and Breazeal (2006a) (implicit/explicit training,^2^ human/machine leading, and human guidance/exploration).
- *Pragmatic frame*: the pragmatic frame used in the learning interaction will be described together with the form/usage/flexibility of the information types this pragmatic frame encompasses.
- *Implicit knowledge*: further, we detail the implicit knowledge given to the robot and the human user and point out what the robot actually does *not* know beforehand.

Whereas, as described in the Introduction, naturally, pragmatic frames emerge in social interaction, in HRI so far they have been most often hard-coded into the system as fixed interaction protocols. The developed learning algorithms often stand in the focus of the research, and thus, the design of the pragmatic frame which the respective studies use is adapted directly to the given algorithm. Since the learning algorithms are therefore often in the center, we will structure our analysis into four categories of learning which regroup Cuayáhuitl’s categories (Cuayáhuitl, 2015) to take the HRI perspective into account:

- *Passive learning*: passive learning refers to the most basic category of interaction for machine learning techniques, in which the robot passively observes the user’s demonstration for learning – as opposed to actively querying the user for information. It includes supervised and unsupervised approaches with symbolic encoding or encoding at trajectory level and interactive programing techniques in which, as opposed to the other machine learning techniques, no abstraction or transcription of data into a new code (and thus, no generalization) is taking place, but data are merely stored.
- *Exploration learning*: in the exploration category, we mainly find reinforcement learning (RL) techniques, which we divide into the following two categories: *with initial tutor demonstration* and *with tutor refinement*. Importantly, these are not mutually exclusive, but there are approaches with both initialization and refinement from the tutor. These two types of approaches are also distinguished for RL in Billard et al. (2008) (Figure 59.18), where they are referred to as approaches with “Self exploration of the learned skill” and approaches with “Refinement of the learned skill through the user’s support,” respectively.
  - *With initial tutor demonstration*: this category of approaches comprises RL approaches that use techniques learning from internal reward functions built after observing human behavior and other approaches also using the tutor’s demonstration as initialization or seed for exploration.
  - *With tutor refinement*: this category includes RL approaches that do not learn *via* optimizing a reward function, but the rewards/reinforcements are given externally, e.g., from a human tutor who is iteratively providing feedback to the robot’s actions.
- *Active learning*: the category of active learning refers to approaches, in which the robot leads the interaction by querying the user’s input. Of course active learning machine learning techniques fall into this category but also techniques that do not issue queries based on any algorithms (according to which the action of the robot is chosen to, for example, maximize the expected information gain from human feedback), such that the queries are preprogrammed or simply systematically cover all training examples.

There are many dimensions the works could be grouped in; we chose the one described above, but they could also be grouped along the dimension of flexibility allowed in the used pragmatic frame for instance. For each of these categories of approaches, we will identify a basic pragmatic frame, which is common to all works of the category and is displayed in Tables 1–4. In addition to the syntactic elements of information, this basic common pragmatic frame depicts when in the interactional pattern learning is taking place.

### 2.2 Analysis

In the following, we present a range of learning approaches and analyze them with respect to the pragmatic frames that they implicitly encode and use in their experimental protocols. These experimental protocols differ from pragmatic frames occurring in natural interactions (1) in that they are predefined (by the programmer) and not evolved through interaction with the teacher and (2) in that they often include an experimenter (often the programmer) who provides the learning system with important cues such as start and end times for recording the learning data, information about unsuccessful teaching trials, etc.

#### 2.2.1 Passive Learning

In this category, we first present works in which users teach the robot interactively sequential tasks with symbolic encoding of sequences of predefined actions. This can be done through several means: verbal commands, a graphical user interface (GUI), and kinesthetic teaching. We also consider interactive programing approaches, even though they merely store input without abstraction, transcription, or generalization, because from the user perspective, the interactions these approaches entail would be considered learning/teaching interactions as well. Lallée et al. (2010), Saunders et al. (2006), and Nicolescu and Mataric (2005) describe respective approaches. Other works we will not analyze here that fall into this passive learning category are, for example, the works of Kuniyoshi et al. (1994), Voyles and Khosla (1998), Ijspeert et al. (2002), Lieberman (2001), and Lauria et al. (2002). The work of Petit et al. (2013), where a human user and a robot cooperate to carry out tasks as, for example, organizing objects into boxes, is similar to the described approaches as well; however, it also falls into the active learning category since the robot uses queries to actively signal unknown actions to the user. Teaching can not only be done *via* speech but also by imitation or kinesthetic teaching. This type of approach could actually be considered a form of learning new frames which are a shared plan to achieve joint actions, but these learned frames are not teaching/learning frames: they are not themselves frames used to teach new words or new actions where the structure is providing cues to help in the statistical inference.

##### 2.2.1.1 Lallée et al. (2010) (Section 2)

###### 2.2.1.1.1 Focus

The focus of the work is to teach sequential tasks in a natural and intuitive way. The authors present an approach they term “Spoken Language Programing,” which uses natural language to give commands to a humanoid HRP-2 robot in a cooperative construction task with a human at a table. The human user and robot have to build a table together. The legs of the table are joined with the tabletop using screws. In the presented approach, no model is built and input is not generalized, but a sequence of already known behaviors is remembered for repeated execution.

###### 2.2.1.1.2 HRI Category

Here, training is explicit. The interaction is lead by the human who guides the robot to assist him/her.

###### 2.2.1.1.3 Pragmatic Frame

The robot signals the start of the interaction with uttering that it is ready (Table S1 in Supplementary Material). The user signals the start of the input, the input, and the confirmation that the input should be stored by means of a sequence of verbal commands. The robot asks to confirm the user’s command after every command. The user gives positive or negative feedback. On positive feedback, the robot executes the respective command and confirms that the sequence can advance. On negative feedback, the robot only gives confirmation and the user can repeat the command. The stored sequence of actions can then be played back. As user and robot are collaboratively working on a construction task, when there are steps on which the robot has to wait for the user to finish his/her action, the sequence can be advanced by the user *via* a “continue” command when the action is completed.

Basic example taken from Lallée et al. (2010) (R robot, U user):

R: I am ready.
U: Learn.
R: You said learn? Yes, I’ll start learning now. I am ready.
U: Prepare.
R: You said prepare?
U: Yes.
R: Preparing to grasp. … I am ready.
U: Left open.
R: You said left open?
U: Yes.
R: Opening left hand. … I am ready.
etc.
U: OK.
R: You said OK?
U: Yes.
R: OK we will store this plan. I am ready.

For subsequent performance:

U: Macro.
R: You said macro?
U: Yes.
R: Running the macro. Preparing to grasp. Waiting for your signal.
U: Continue.
etc.
R: In line macro finished. I am ready.

###### 2.2.1.1.4 Implicit Knowledge

The robot is supplied with three text files for speech recognition (object names, posture names, and behavior names), a set of atomic action primitives, and the available control commands. It knows how to parse the input and what to do in the sequence. The user knows the robot’s atomic action primitives; the control commands; and objects, postures, and behaviors. The robot does not know beforehand the sequence of basic actions it should perform. Also, it can be taught the correspondence between a label and a perceived object and actions additional to the set of basic action primitives, which can be hierarchically combined to form new actions.

##### 2.2.1.2 Saunders et al. (2006)

###### 2.2.1.2.1 Focus

This work focuses on an intuitive method to construct state/action memory maps in a hierarchical manner by “molding” and “scaffolding.” The human user teaches a small 5-cm diameter Khepera mobile robot with vision sensor and gripper in a maze-like environment on an office desk *via* a screen-based GUI. For moving around the environment with different objects and containers, the user teaches the robot tasks on three levels: (1) sequences of known primitives, (2) tasks with a goal state, and (3) behaviors (the two latter depending on the environmental state). The user is basically programing the robot in an interactive manner by controlling the robot remotely using a screen-based GUI. For instance, a behavior the user could teach the robot is to move forward, when a light is off and backwards when a light is on avoiding obstacles. The resulting built task hierarchy of *behaviors* consisting of tasks, sequences, and primitives (*tasks* consisting of sequences and primitives, and *sequences* consisting of primitives) corresponds to an action selection mechanism based on a simple k-nearest neighbor approach: when performing what the robot has learned, the decision of which action to execute next is based on the robot’s current state (IR sensors, distance to light, angle to light, is the gripper open?, etc.).

###### 2.2.1.2.2 HRI Category

Teaching or programing the robot is explicit in this work. The user is guiding the robot *via* the programing interface and leads the “interaction.”

###### 2.2.1.2.3 Pragmatic Frame

The interaction presupposed in this approach is rather unidirectional (Table S2 in Supplementary Material). Apart from executing commands given *via* button presses, the robot is not further involved in an interaction. The user is operating the robot from the computer (molding), and this is a special feature and can modify the training area of the robot for a higher information gain (scaffolding). The flexibility of information mainly lies in the aspect that, similar to other visual programing tools, such as Choregraphe by Aldebaran (Pot et al., 2009), the exact program (which parts to implement first or how the program is realized) is up to the user. Additionally, it is possible to run individual segments of code separately. The user thus gives the learning input including when it starts and ends, and commands the execution of movements (learned or primitives). As there are buttons for every command, the user has no flexibility in the form of information except the constellation of commands forming the program.

###### 2.2.1.2.4 Implicit Knowledge

The robot has a predefined set of action primitives and relevant sensors for the given task environment. It also knows what each button press from the user interface means and the rules for executing the learned actions. The robot does not know the program beforehand. The human user should know the meaning of buttons and how the robot works. The set of robot action primitives and what each level of teaching (sequence, task, and behavior) entails should be given to the user. Moreover, the fact that the user is programing the robot requires the user to come up with a plan for realizing the program whose complexity is proportional to the complexity of the task to be taught and of the robot. Therefore, the proposed method might be difficult for non-expert users to use even if 100% familiar with the robot sensors and action capabilities.

##### 2.2.1.3 Nicolescu and Mataric (2005)

###### 2.2.1.3.1 Focus

The Pioneer 2-DX mobile robot in this work is equipped with sonars, laser range finder, a camera, and a gripper. It learns representations of high-level sequential navigation/manipulation tasks in a 5.4 m × 6.6 m arena. This is done by building a graph-based behavior network from the demonstrations by a human user with a head-set for voice recognition who is guiding the wheeled robot that follows step-wise through a maze. Hereby, sequences of predefined basic behaviors are built. These basic behaviors are represented as nodes in the graph. Multiple demonstrations can be merged by computing the longest common subsequence of their nodes.

###### 2.2.1.3.2 HRI Category

Teaching is explicit in this example. The interaction is lead by the user who guides the robot.

###### 2.2.1.3.3 Pragmatic Frame

The user provides guidance and verbal commands bearing some flexibility: the first phase of the interaction is the learning phase (Table S3 in Supplementary Material). The user signals start and end of the demonstration by saying the verbal commands “start” and “end.” He or she walks through a maze and gives commands to take or drop objects. Optionally, he or she can also give a signal (i.e., say “here”) to direct the robot’s attention in order to disambiguate the task. In the second phase, during the robot’s performance, the user can give corrective feedback online and delete additional or insert missing elements of behaviors by saying “bad” or “come … go” and showing the behavior that should be inserted. The second phase for performing the action presents another separate pragmatic frame in which learned knowledge can be altered in the communicative cognitive operation of deleting and inserting behavior elements. The function of inserting behavior elements is equal to the adding knowledge function of the learning frame in the first phase. This learning frame thus is embedded in the performance frame with its knowledge retrieval communicative cognitive function. We would like to remark at this point that pragmatic frames can be hierarchically nested and this interaction structure is a simple example of this.

###### 2.2.1.3.4 Implicit Knowledge

For this task, the robot knows beforehand about the predefined set of verbal user commands and how to follow the user by detecting legs. It knows the sequence underlying the pragmatic frame and how to detect and order the basic behaviors. The robot is not aware of the sequence of behaviors and the resulting path through the arena. The user also is aware of this syntax of the pragmatic frame including the rules of each of the two phases. He or she knows the set of robot behaviors, the robot’s sensors, and all spoken commands possible and their meaning.

Additionally to the above approaches, this category comprises, on the one hand, works with classifiers trained on (hand-)labeled data provided by the user or experimenter, or works learning movements with neural network models trained in a supervised manner using a human teacher’s demonstrations (backpropagation through time) and, on the other hand, works learning from unlabeled data, representing movement probabilistically (e.g., with GMMs).

The approaches presented by Thomaz and Cakmak (2009) and Yamashita and Tani (2008) represent two different supervised learning mechanisms.

##### 2.2.1.4 Thomaz and Cakmak (2009)

###### 2.2.1.4.1 Focus

This work presents an interaction for affordance learning in which a small humanoid robot torso, Junior, is shown different objects by a human user. The authors are interested in investigating how humans teach, how the robot can influence the teacher, and the resulting impact on machine learning algorithms.

###### 2.2.1.4.2 HRI Categories

With respect to the HRI categories, the interaction is led by the human who guides the robot in a type of preprogrammed exploration. Teaching is done explicitly in this example.

###### 2.2.1.4.3 Pragmatic Frame

The user presents one object at a time and positions it centered in the robot’s field of view (Table S4 in Supplementary Material). Once the robot detects the object, it performs one of the two predefined actions: single arm swing and two arm grasp. If the robot does not recognize any object (it is too close or too far), it tilts its neck to the upper limit to indicate an error. The user then should reposition the object. After the interaction with the user, the affordances are hand-labeled by the experimenter and support vector machine (SVM) classifiers are trained offline. The frame allows flexibility for the user who chooses which object to present and how many times. Information passed from user to robot (i.e., the positioning of the object) is used for learning and information from robot to user (i.e., the error indication signal) is used for transparency of the robot system.

###### 2.2.1.4.4 Implicit Knowledge

Knowledge which is given to the robot implicitly includes to look for predefined objects and how to detect/distinguish them (based on color), which action to perform, and the sequence of actions in the frame. Additionally, the learning algorithm needs tuples [initial object state (distance; orientation); action; affordance (hand-labeled)] as input. The robot thus does not know beforehand how to predict the outcome (or rather the object affordance) when a certain action is performed given an initial object state. The human user is aware of the sequence and rules of the interaction and the robot’s behavior (except the neck tilt in case of an input error and how to react).

##### 2.2.1.5 Yamashita and Tani (2008)

###### 2.2.1.5.1 Focus

In this work, a small humanoid robot manipulated a 9 cm × 9 cm × 9 cm cubic object on a workbench in front of it. It should learn to reproduce five different behaviors (one of four different simple object manipulations, such as moving the object left and right three times or clapping of the hands) from kinesthetic demonstrations. The authors present a neural network model for learning whose weights are optimized to represent the data by comparing the model output to the goal action shown through kinesthetic teaching. The focus of this work lies clearly on the modeling technique, whereas the interaction between user and robot is disregarded.

###### 2.2.1.5.2 Pragmatic Frame

In such an interaction, the sole information from the user to the robot would be the kinesthetic demonstrations with which the human guides the robot (Table S5 in Supplementary Material). For each task, the robot is presented with demonstrations for five different object positions. The experimenter would provide all other information to the robot and is leading the interaction. There is thus no flexibility at all in any of the information types.

###### 2.2.1.5.3 HRI Category

A hypothetical interaction is characterized by human guidance, and the experimenter leads the interaction. This could be called explicit teaching, but users could also only provide the training data without having been told and thus having the intention to teach.

###### 2.2.1.5.4 Implicit Knowledge

With respect to implicit knowledge, the robot knows to visually track the object and record encoder values for each joint as learning input. It does not know the trajectories beforehand and generates a compact quasi-symbolic representation of data by identifying common parts of different task trajectories that are encoded by single neurons. Everything else is provided *via* programing by the experimenter/programmer. The user here is only providing the input; this is rather a batch learning approach feeding pre-recorded input data into the system without any interaction.

Calinon et al. (2010), Mühlig et al. (2012), and Akgun et al. (2012) present approaches to learning motor skills using probabilistic movement representations.

##### 2.2.1.6 Calinon et al. (2010) (Section VI)

###### 2.2.1.6.1 Focus

The authors describe an experiment in which a Fujitsu HOAP-3 humanoid robot is kinesthetically taught to feed a Robota doll by first bringing a spoon in the robot’s hand to a plate with mashed potatoes and then moving it to Robota’s mouth. The focus of the work lies in learning a controller with several constraints (i.e., multiple landmarks) and the generalization capabilities of the approach. Their learning framework trains a controller relative to two landmarks (the plate and the mouth of the doll that is linked to the robot’s kinematic model) for reproduction of the movement shown in four kinesthetic demonstrations with varying positions of the landmarks. The movement is reproduced *via* the combination of hidden Markov models (HMMs) for each landmark encoding the relative trajectories and Gaussian mixture regression (GMR), such that the robot satisfies the constraints in order to reproduce the movement in a new situation (different landmark positions or perturbations).

###### 2.2.1.6.2 HRI Category

The interaction in which the human guides the robot is lead by the experimenter. Also for this work, this could be called explicit teaching, but users could also only provide the training data without having been told and thus having the intention to teach.

###### 2.2.1.6.3 Pragmatic Frame

The start and end of the recording of the movement is given to the robot by the researchers (presumably through pressing a button or the like) (Table S6 in Supplementary Material). Thus, the components of the interactional sequence employed in this example are the start of the movement, leading the arm of the robot through the movement (from the home position to landmark 1, plate, to the goal, landmark 2, mouth of the doll), and finally the end of the movement. The user’s role is solely to provide kinesthetic demonstrations of the movement and is not further involved in an interaction. The interaction would follow the pragmatic frame shown in Table S6 in Supplementary Material and bears no flexibility.

###### 2.2.1.6.4 Implicit Information

The robot is given the information when the trajectory it should record begins and ends by the experimenter. It knows that for learning, it should pay attention to the trajectory itself and not its end position or end state, and the robot knows that and how it should track the two landmarks and how to represent the trajectory. The robot does not know the exact trajectory beforehand.

##### 2.2.1.7 Mühlig et al. (2012) and Gienger et al. (2010)

###### 2.2.1.7.1 Focus

In the work presented in these papers, the human tutor is sitting at a table with different objects and demonstrates tasks to a Honda humanoid research robot (e.g., how to stack two objects or how to pour a beverage). The robot witnesses the demonstrations from a small distance away from the table and walks up to the table to perform the movement itself. The authors of this work put the emphasis on the interaction between user and robot and the generalization capabilities of the learning approach.

###### 2.2.1.7.2 HRI Category

The interaction is led by the human in a human guidance imitation learning interaction. In an interaction with users other than the developer/experimenter himself, teaching here would be explicit.

###### 2.2.1.7.3 Pragmatic Frame

In a pick and place scenario, the user guides the humanoid robot and provides most information to the robot *via* predefined artificial signals (e.g., lifting one or two hands in a fist) upon which the robot signals the receipt of information through gaze (Table S7 in Supplementary Material). The movements are learned with Gaussian mixture models. In this work, the robot detects the start and end of the trajectory itself. At first, the robot gazes toward the most salient object. The user can direct the robot’s attention to the relevant objects by touching them. The user then presents the demonstration of the movement. Upon its detected completion, the robot gazes at the tutor who then gives a signal to store the demonstration by lifting the left hand in a fist. At this point, the robot again shifts to a saliency-based gaze while the user moves the objects to the robot’s side of the table and lifts either one or both hands to prompt performance of the learned movement with one or both hands, respectively. At this point, the robot informs the human of possible predicted difficulties for carrying out the movement. The human in this case either aborts or confirms the execution. The robot walks up to the table, grasps the relevant objects, and imitates the movement.

###### 2.2.1.7.4 Implicit Knowledge

Thus, the robot knows beforehand which objects can be involved in the demonstration and how to detect its start and end. It knows the overall sequence with all signals and how to detect them, as well as how to represent the movements (predefined feature points for each known object, choice of task space based on lowest inter-trial variance between demonstrations). The robot does not know beforehand which objects are involved in the demonstration, what to do with them (i.e., the movement trajectory), and if it should reproduce the movement with one hand or both hands. The user should know the rigid behavior pattern of the pragmatic frame as well as all signals.

##### 2.2.1.8 Akgun et al. (2012)

###### 2.2.1.8.1 Focus

The upper torso humanoid robot, Simon, learns goal-oriented and means-oriented movements, such as inserting a block through a hole, stacking a block on top of another, performing a beckon gesture asking someone to come closer, or raising the hand, in an unsupervised manner comparing trajectory and keyframe kinesthetic demonstrations. We here consider the latter as they can be corrected step-wise. In this type of teaching, the human user moves the robots arm and picks configurations as keyframes by saying “Record frame.” The user in general gives commands to the robot *via* speech.

###### 2.2.1.8.2 HRI Categorization

Concerning the HRI categorization, training here is explicit. The interaction is lead by the human who guides the robot.

###### 2.2.1.8.3 Pragmatic Frame

Similar to the approach presented in Nicolescu and Mataric (2005), in this work, two pragmatic frames are involved in the learning/teaching interaction (Table S8 in Supplementary Material). The first pragmatic frame concerns storing the information, and in the second frame, this information is accessed and modified *via* speech signals. “Next frame” and “previous frame” let the user navigate through the previous demonstration; when saying “modify this frame,” the user can move the robot’s arm to a new configuration and thereby modify the former frame, add a new frame after the current one with “add new frame,” and delete the frame after the current one with “delete this frame.” The user can retrieve the resulting demonstration with the command “play current demonstration” and if satisfied with it, submit it as a new instance to the learning set (command: “record this demonstration”). The step-wise correction allows for a certain flexibility of demonstration (number and location of keyframes, correction). This 2-stage teaching with correction or refinement is also possible in other approaches (e.g., Calinon and Billard (2007), Lee and Ott (2011), and Kormushev et al. (2011)).

###### 2.2.1.8.4 Implicit Knowledge

Yet, user and robot must know the sequence of actions underlying the pragmatic frame as well as the predefined verbal commands through which the user provides information to the robot. The robot also knows which sensors to record and to pay attention to the trajectory, as opposed to the end state of the action. The only information that the robot is not given beforehand is the exact movement in terms of keyframes. Some elements of the interaction are optional, and the decision to enter into a certain element is up to the user who can also decide how many demonstrations he/she gives.

For these passive learning approaches, comprising the interactive programing approaches, the basic common pragmatic frame we identified is shown in Table 1. The user provides the learning input to the robot, which then learns from the data and is optionally performing the learned task.

#### 2.2.2 Exploration Learning with Initial Tutor Demonstration

The exploration learning category of approaches comprises approaches that use techniques learning with an initial tutor demonstration, which we will describe first. Grollman and Billard (2011) present an approach (which does not use RL) belonging to this category and in principle (Lopes et al., 2007) also falls into this category but is a special case such that its frame structure rather reflects to be part of the passive learning category, which we will detail below. Other works we will not discuss that also belong to this category are, for example, Atkeson and Schaal (1997) and Smart and Kaelbling (2002).

##### 2.2.2.1 Grollman and Billard (2011)

###### 2.2.2.1.1 Focus

The authors present an approach for the procedural learning of a movement skill. Their system (using a Barrett WAM robotic arm) learns from failed kinesthetic demonstrations to flip up a styrofoam block to stand on one end on a table or to play basket ball with a catapult. Movements are represented with GMMs.

###### 2.2.2.1.2 HRI Categories

The authors do not describe an interaction with a user or a user study, and therefore, HRI categories are difficult to determine. Teaching is not necessarily explicit. In any case, the approach is positioned rather on the side of exploration than human guidance.

###### 2.2.2.1.3 Pragmatic Frame

The user only provides two specific failed demonstrations (with not enough and too much momentum) to the robot as learning input (Table S9 in Supplementary Material). In a hypothetical interaction, the user would not have any flexibility to present, as here input demonstrations are specifically chosen to match the criteria of the learning algorithm. In an interaction, this selection step would most likely remain because the task is also difficult for humans to perform, and the result is not easily controllable. Here, the experimenter is responsible for the information of the start and end of the demonstrations and their selection and possible preprocessing.

###### 2.2.2.1.4 Implicit Information

The robot knows what the input means and also what is important about the movement: in its exploration, its movements should agree on start and end positions of the demonstrations. It should reproduce agreements with respect to maximum velocity and timing of the two demonstrations and explore on disagreements. The robot only does not know the demonstrations beforehand.

The basic pragmatic frame of exploration learning approaches with initial user demonstration is depicted in Table 2.

##### 2.2.2.2 Lopes et al. (2007)

###### 2.2.2.2.1 Focus

This work presents a special case for the “Exploration learning” category, in which sequential task demonstrations from a human user are recognized and the task is learned based on an object affordance-based world model. Without interaction with a user, the robot first learns affordances with Bayesian networks providing it with the world dynamics of the setup. Second, the user demonstrations are interpreted in terms of the robot’s own action repertoire and thus can be reproduced by extracting the reward function *via* Bayesian inverse reinforcement learning and computing the optimal policy. Importantly, in this example, due to the known world model, the robot does not explore in order to obtain the optimal policy after having learned the reward function. Instead, the optimal policy is computed.

###### 2.2.2.2.2 HRI Category

The training in this example could be implicit or explicit as the robot only passively observes the user’s demonstrations. Therefore, no real interaction is taking place.

###### 2.2.2.2.3 Pragmatic Frame

The robot platform BALTAZAR, a torso with one arm, first learns the affordances of three type of objects (i.e., large balls, small balls, and boxes) by exploration with the three actions it has in its repertoire (i.e., grasping, tapping, and touching) (Table S10 in Supplementary Material). The user comes into play after this and demonstrates what to do in a “recycling game” in which objects of different size, shape, and color have to be separated with different actions: boxes should be dropped into a container, small balls should be tapped off the table, and the top of large balls should be touched whereupon the ball is removed from the table by the experimenter. The workspace of the robot consists of two positions (left and right), at which each action can be performed. The user provides demonstrations with actions for all possible states of the state-space (16 states equal to the number of possible combinations of objects at the two positions plus one additional state for when the robot’s actions fail). The system is able to cope with a certain degree of incompleteness and inaccurateness of the demonstrations. The robot classifies the actions of a demonstration according to the observed effects, corresponding to the known affordances (i.e., the before built simple world model), learns a reward function (with which an optimal policy is computed using the learned world model), and performs the actions itself when presented with the respective initial state.

###### 2.2.2.2.4 Implicit Knowledge

The robot has three available action primitives: it knows the features according to which to classify the objects and how to describe the effects of actions on the objects (velocity, contact, object-hand distance). The affordances are not known beforehand but are learned with about 250 trials of acting on one of the objects (Montesano et al., 2007). The order of objects and the number of trials for each of them is determined by the experimenter. For the second part of the work, the robot does not know the rules of the recycling game beforehand (i.e., it does not have the reward function or optimal policy). The human user is presented with each initial state by the experimenter and performs according to the policy of the recycling task.

#### 2.2.3 Exploration Learning with User Refinement

In this part, we present exploration learning approaches, where the actions of the robot are iteratively refined with the user’s input (e.g., feedback or guidance). Kaplan et al. (2002), Grizou et al. (2013, 2014), and Steels and Kaplan (2002) present respective approaches but also the approaches described, for instance, in Blumberg et al. (2002), Lockerd and Breazeal (2004), Isbell et al. (2001), and Kuhlmann et al. (2004) fall into this category.

##### 2.2.3.1 Kaplan et al. (2002)

###### 2.2.3.1.1 Focus

The authors implement a technique used to train dogs called clicker training to teach the AIBO robot sequences of actions, as moving in a clock-wise circle, for example. Defining reinforcement signals for the robot, the user gives feedback as positive reward after an observed correct action. No reward function is defined in this technique, which is a form of shaping (Saksida et al., 1997).

###### 2.2.3.1.2 HRI Category

Thus, the human leads the interaction in explicit training, which incorporates both human guidance and robot exploration.

###### 2.2.3.1.3 Pragmatic Frame

In the first phase, the user teaches a secondary reinforcer by presenting it in conjunction with a previously defined positive signal as a primary reinforcer (Table S11 in Supplementary Material). After having witnessed these two reinforcers together many times, the robot has learned and confirms the secondary reinforcer. In the second phase then, the robot shows an exploratory behavior based on a control architecture. The user gives positive feedback in form of the secondary reinforcer upon a correct action the robot performs until the robot can put together the whole desired target sequence. This is confirmed by the user with the primary reinforcer and a label is presented to name the learned sequence. The robot confirms the storage of the label. Then, the robot is able to perform the learned sequence and if no positive feedback is provided can alter this sequence slightly in another exploration loop. To summarize, apart from the explorative actions, the robot gives signals to the user in order to confirm the secondary reinforcer and the receipt of the input in form of a label for the learned sequence (wagging its tail or blinking its eyes). The confirmation signals serve as transparency device of the system, but the work does not detail what is the user’s role in case of an error situation in which this confirmation signal is absent (if the robot for instance fails to detect the input). Without confirmation signal for the secondary reinforcer, the robot behaves according to the absence of a secondary reinforcer which means to the robot that it is not getting closer to the behavioral goal, which is used by the robot to redirect its exploration in other directions. Thus, the previous behaviors might have to be repeated by the user. The exact form of the secondary reinforcer is up to the user (possibilities range from choosing a visual stimulus to choosing a verbal command). The user also chooses the label of the target sequence.

###### 2.2.3.1.4 Implicit Knowledge

The robot is looking for a predefined primary reinforcer and knows from preprogrammed rules how to detect the secondary reinforcer (within 5 s before the primary reinforcer, 30 times). It is given all verbal commands, the pre-programed high-level behaviors, the exploration rules, as well as the behavior sequence of the pragmatic frame. It does not know beforehand the form of the secondary reinforcer and what the task is. The user is aware of the possible primary reinforcers which are chosen by the experimenters; he or she knows how clicker training works and the exact sequence of the pragmatic frame, the verbal command for starting the exploration (“try”) and the transparency signals.

##### 2.2.3.2 Grizou et al. (2013, 2014)

###### 2.2.3.2.1 Focus

The authors take the first step to provide the user with more flexibility. The two papers present two different scenarios, in which a sequential task and at the same time a feedback-to-meaning mapping is learned. In the first paper, a robotic arm with a gripper learns how to stack 3 blocks in towers of up to 2 blocks onto 4 possible positions in a pick and place scenario. The second paper presents a simulation experiment using EEG data of brain signals elicited by either correct or erroneous assessments used as binary feedback. An agent that can perform the five different actions of moving in either direction and staying at the current position learns which position is the target is should reach in a 5 × 5 grid world with 25 discrete positions and respective 25 possible tasks. In each of the scenarios, the user gives feedback or instructions for each move the learner makes. Technically, the authors represent the task with Markov decision processes. They define a pseudo-likelihood function, computing the likelihood that the system’s prediction about what the meaning of the feedback signals is according to a certain task hypothesis, is equal to what the classifier trained on the signal-meaning pairs will predict for a new situation.

###### 2.2.3.2.2 HRI Category

In this explicit teaching interaction between the robot system and the user, the human provides feedback to the exploration of the robot (when providing binary yes or no feedback), but he/she can also provide input in form of guidance signals (e.g., up, down, left, right, no move) upon which the robot acts. Again, the human guides the robot in interaction which involves either the user leading by providing guidance signals dependent on the robot’s state or the user providing feedback for the robot’s exploration (the robot leads).

###### 2.2.3.2.3 Pragmatic Frame

The form of the verbal commands which represent the feedback or guidance signals can be chosen by the user (Table S12 in Supplementary Material). This approach is special in the sense that part of the pragmatic frame is learned. The system does not know which signal from the user corresponds to which given meaning. We think that this mapping is part of the pragmatic frame itself. Thus, this approach is the sole example among the presented papers, where part of the pragmatic frame is learned.

###### 2.2.3.2.4 Implicit Knowledge

The robot and user know the feedback signal features, which actions the robot can choose, the set of possible tasks, the set of meanings, and the sequence of actions of the interaction (especially the timing). The robot does not know beforehand the mapping of feedback and guidance signals to their meanings, and it does not know the task in terms of which it is the goal position.

##### 2.2.3.3 Steels and Kaplan (2002)

###### 2.2.3.3.1 Focus

Whereas in the previously described work, the exact words used to teach the robot could be variable (open-ended variability with the limit being detection capabilities), the authors of this work allow for a certain variability in the dialog surface structure of how the learning input is provided. The task the robot AIBO should learn is about image-label associations. The authors aim at showing that these can be learned in social interaction with a human user under varying lighting conditions.

###### 2.2.3.3.2 HRI Category

The user leads the interaction and guides the robot in acquiring object labels in this explicit learning situation.

###### 2.2.3.3.3 Pragmatic Frame

The object (such as a red ball or a similar toy) is always presented by the user who can then either give directly the label to the robot (“Ball.”), ask the robot to produce a label (“What is it?”), or provide either a correct (“Is it … Ball?”) or an incorrect label (“Is it … Smiley?”) to the robot that can then correct the user (“No; ball.”) (Table S13 in Supplementary Material). The robot signals attention to the object by looking at it and trying to touch it. Then, it produces the learning input (i.e., the label) itself in all three possible subsequences (“Ball (?)”) and receives a verbal feedback with potential correction from the user (“Good.”, “Yes.”, “No; listen; Ball.”), which is used for learning. This is another example, where there is not only one pragmatic frame used. Whereas the form of the dialog to provide the robot with the label implies no difference for the learning mechanism (as the association of the produced label with the visual perception of the object is strengthened or weakened according to the user’s feedback in all cases), from the perspective of communicative cognitive functions, the robot either adds knowledge, retrieves acquired knowledge from memory, or compares a given label with the respective knowledge in memory. Thus, we consider this approach to involve three different pragmatic frames.

###### 2.2.3.3.4 Implicit Knowledge

The robot has the implicit knowledge to look for objects and words as input and how to find it: the robot is supplied with a lexicon for speech recognition, and it knows how to detect three predefined objects. It is also aware of the sequence of the pragmatic frame. The robot does not know which label corresponds to the objects it detects. The user is instructed about the pragmatic frame and which words to use (including to keep the input simple and say “Ball” instead of, for example, “This is a pretty red ball that is for throwing and dogs will catch it.”).

For the pragmatic frames of the presented approaches using exploration learning with user refinement, we identified a common basis where the user gives feedback (binary feedback or guidance signals) on the robot’s exploration from which the system learns (see Table 3).

#### 2.2.4 Active Learning

The active learning category comprises approaches in which the robot leads the interaction by querying the user’s input. Of course active learning machine learning techniques fall into this category but also techniques that do not issue queries based on any algorithms.

##### 2.2.4.1 Calinon and Billard (2006)

###### 2.2.4.1.1 Focus

In this work, communicative gestures are learned in a supervised manner through an imitation game. The focus lies on speeding up the convergence of the used learning algorithms by exploiting social cues from the human teacher. The first phase of the interaction consists in a game, in which a small Fujitsu HOAP-2 humanoid robot displays different pre-programed communicative gestures (such as pointing to an object, mutual gaze, or a turn-taking signal) and the user who is sitting opposite the robot at a table imitates them. This is the actual learning part of the interaction. In the second phase of the interaction, the previously learned gestures serve as social signals, when the user shows pointing-at-objects gestures to the robot that should recognize the target object and subsequently point to the same object.

###### 2.2.4.1.2 HRI Category

In this example, the machine leads the interaction by acting first and prompting the user to act, even though the overall aim is to be able to deal with person specific characteristics of gestures. However, the human guides the machine in explicit teaching, as there is no exploration involved and the human user provides the learning input.

###### 2.2.4.1.3 Pragmatic Frame

In the imitation game, in the first phase, the user can decide how often he/she wants to produce a certain action (Table S14 in Supplementary Material). In the second phase, gaze plays the role of displaying attention to the user’s gesture and also functions as a turn-taking cue, prompting performance or feedback by mutual gaze. The user can correct the robot by showing the previous gesture again.

###### 2.2.4.1.4 Implicit Knowledge

The implicit knowledge provided to the robot comprises the sequence of actions of the pragmatic frame, to record data from the X-sens motion sensors with a correspondence of joint angles and robot DOFs, and the gestures and their meaning. Additionally, there is a calibration phase prior to the experiment, in which object locations are stored and kinesthetic recordings of the communicative gestures are provided. It remains unclear how the robot segments the movement from the user recorded with the motion sensors. The robot does not know beforehand the way the human executes the gesture. The human user must know about his or her role during the phases of the interaction and the sequence of the frame together with possible (gaze) cues.

##### 2.2.4.2 Cakmak and Thomaz (2012)

###### 2.2.4.2.1 Focus

In this work, no learning algorithm is involved, because the focus lies on determining the human users’ preference for robot questions. In theory, the work involves kinesthetic trajectory learning of tasks such as pouring cereal into a bowl, adding salt to a salad, or pouring soda into a cup.

###### 2.2.4.2.2 HRI Category

The interaction is led by the machine that asks specific pre-scripted questions about the demonstrated task and, with respect to this point, controls the interaction. The human user gives demonstrations as learning input in explicit teaching with which he or she guides the robot.

###### 2.2.4.2.3 Pragmatic Frame

The user gives two kinesthetic demonstrations of a specific task, including telling the robot verbally when a demonstration starts and ends (“New demonstration” and “End of demonstration”) (Table S15 in Supplementary Material). The robot confirms the commands with speech and head nods. It gazes to the object during the demonstration. Again these signals serve as transparency signals, but the authors do not describe what are the options in case of a transparent error. The user then asks the robot if it had any questions to which the robot responds with one of two pre-scripted queries for each of the demonstrated actions: for the task of pouring cereal into a bowl, the robot asks a query to determine if a certain way of executing the task is also acceptable (tip the box of cereal over too early or approaching the bowl from an uncommon direction). For the task of adding salt to a salad, the robot asks a query by first creating a new scenario and requesting a new demonstration from the teacher (starting from a position either slightly outside the expected range or high above the bowl). For the task of pouring soda, the robot asks a query about a certain feature of the demonstration (such as a certain orientation at the start of the movement or the importance of the height from which to pour). The user answers the query verbally after which the sequence is advanced through a button press by the experimenter. As there is no learning involved, the query answer does not have any function and is not used for learning or as feedback. In this work, the authors assessed the participants’ preference for the type of queries by evaluating the time it took participants to answer the query and a questionnaire on the perceived smartness, informativeness, and ease of the question. The user’s exact verbal response is not predefined and thus is variable in its form, although the modality of response is fixed.

###### 2.2.4.2.4 Implicit Knowledge

The robot is given the set of pre-scripted queries (two for each of the three tasks) and the sequence of the pragmatic frame according to which it behaves. The user is instructed about the sequence of the frame using a video example of an interaction involving a similar task. He or she knows the possible speech commands with a reminder on the nearby whiteboard and if he or she does not know how to respond to certain queries, the experimenters would help.

For the active learning approaches, we identified the basic common pragmatic frame as shown in Table 4. The robot leads and asks the user for a certain input which the user provides. The robot learns from this and optionally performs the learned task.

### 2.3 Discussion

The following discussion represents a synthesis of the above analyses. In general, for the presented pragmatic frames, we find only little flexibility of information types. The structure of the frames is rigid and freedom is not often granted to users as deviations most likely cannot be dealt with by the robot system and lead to errors. A naive user thus has to be familiar with this pre-programed artificial sequence and adeptly perform its restricted role without errors. For many of the above papers, especially the approaches involving exploration learning with initial tutor demonstration and some passive learning approaches, the user even assumes a minor role as the input producer and no real interaction is established. These works do not reveal the situation (including who is performing, the detection, segmentation, and even preprocessing of data, potential instructions of users, etc.) in which input is collected. In most papers, as the focus is another, the programmers themselves record and prepare the learning input and feed it into the system.

Concerning the usage of information types, in most works, the robot performance can be probed but the acquired knowledge cannot be modified. Similarly, most signals the robot gives serve transparency of system processes, but in most works, these are not put to good use as there is no workflow/consequence for the user in case of erroneous or missing processing. It seems that the programmer or adept experimenter in such a situation must abort the interaction and learning process.

All reviewed works have in common that they use predefined pragmatic frames with very rigid and mostly artificial structures. Grizou et al. (2013, 2014) present a small exception to this, as in their work, a very first step is taken toward learning a small part of a pragmatic frame. All works use one (for the cognitive communicative function of learning/adding knowledge) or only few (for the cognitive communicative functions of learning, and retrieving, comparing, or altering the acquired knowledge) pragmatic frames and are implicitly assuming much prior knowledge in robot learner and human teacher. These fixed sequences used in the learning approaches presented above imply limits in (a) learning capabilities and (b) interaction, which extend to other works:

(a) Providing this fixed sequence with its matching specifications of the learning algorithm (equivalent to artificial memory processes) to the robot means specifying the relevant parts for learning as implicit knowledge. Thus, learning is restricted to some tasks within a specific setting. In the case where multiple tasks can be learned in one frame, learning is slowed down considerably by statistical inference and could be quicker if richer information about the social interaction could be exploited (cf., Cederborg and Oudeyer (2013)). Consider the example of a common state-of-the-art imitation learning approach we presented above: in Calinon et al. (2010), the authors describe an experiment in which a humanoid robot is taught to feed a doll. The number of pragmatic frames and operations is 1 (see Table S6 in Supplementary Material). Without specifying the goal and what the intention of the tutor is to the robot beforehand, the robot in this example would be faced with a combinatorial explosion. The system coping with an enormous search space would only be able to learn slowly (statistically; distinguishing relevant from irrelevant information). Open-ended cumulative learning using several frames actively, which conveys different forms of information, could accelerate considerably the speed of learning.

Hence, a certain structure in interaction seems beneficial, even necessary for learning. However, this structure should be flexible, and negotiated and learned in interaction with the user, such that the system can cope with multiple pragmatic frames to process not only one kind of learning input.

(b) The sequences also impose heavy constraints to the interaction. An untrained, naive user teaching the robot would most likely not conform to the constraints given by the strictly enforced pragmatic frame, which can even contain artificial signals. When teaching a robot, humans intuitively try to use a range of different interaction frames (cf., Amershi et al. (2014) for an overview). In the example, some users might focus on how to grasp the cutlery and maybe even point out some object properties of cutlery and plate. When showing the action, they might emphasize the manner of the action which is linked to the object used. For instance, a spoon transporting a piece of apple needs to be kept straight during the motion, whereas when using a fork, this is not important. The user might even show the robot a motion that s/he deems wrong in order to give a negative example. Without the adequate frame, the robot could not learn the action from these examples. Thus, the human tutor needs to operate within rigid constraints, if a single predefined frame is used, and these do not admit of interactive freedom or flexibility. Relevantly, artificially designed pragmatic frames imply high costs of teaching.

In contrast to the hard-coded pragmatic frames we find in the robot learning from a human tutor literature, natural human interaction is highly dynamic and flexible, and learning is most certainly not limited to only one single task. In such natural, rich teaching scenarios, it has been shown that important pragmatic structure (pragmatic frames) is provided by the interaction and that this emergent structure is presumably indispensable for comprehending language (Schumacher, 2014) and action (Koterba and Iverson, 2009), as well as for more generalizable learning performance (Thom and Sandhofer, 2009).

We here thus make two major points for pragmatic frames in natural social interaction. These are stability of interaction on the one hand and flexibility of interaction on the other. These two benefits seem to be contradictory at first, so we will try to elucidate this incoherence. Regarding the stability of interaction, pragmatic frames provide a very stable structure with which a learner is presented by the teacher enabling the learner to understand the situation, his/her own role, and pick up and learn the learning content rapidly. However, the structure of pragmatic frames is stable only locally in time. If we zoom out with respect to the time scale point of view (to, for example, a time frame of 1 year), importantly, pragmatic frames emerge and evolve by negotiation between the interaction partners. The flexibility of interaction thus refers to the ability to learn, use, and develop pragmatic frames over time, such that there are multiple pragmatic frames for teaching and learning. We aim at showing the flexibility of natural pragmatic frames by means of their social emergence with presenting the ontogenetic development of a pragmatic frame along a child’s age and capabilities. As mentioned earlier, Bruner (1983) (p. 78 ff.) analyzed the development of the book-reading pragmatic frame in adult–child interaction. In Table 6, we show a respective table for this natural human–human interaction, created with the same tool of analysis we applied for the robot learning literature.

**Table 6:** **A book-reading frame in adult–child interaction (Bruner, 1983)**.

Teaching parent actions	Child learner actions
**Frame 1A**	
1. Direct attention: **optional**, **form**, point and/or verbal command “Look!”	2. Attention: **form**, gaze to image of joint attention
3. Prompt performance: **form**, “What’s that?”	4. Act: **form**, babble strings and smiles
5. Binary feedback + input: **form**, positive feedback and label, “Yes, a fish!”	6. Act: **form**, babble strings and smiles
7. Binary feedback: **form**, positive feedback, “Yes”	
**Frame 1B**	
1. Direct attention: **optional**, **form**, point and/or verbal command “Look!”	2. Attention: **form**, gaze to image of joint attention
3. Prompt performance: **form**, “What’s that?”	4. Act: **form**, lexeme-length babble or words
5. Binary feedback: **form**, positive feedback, “Yes”	
**Frame 1C**	
1. Direct attention: **optional**, **form** point and/or verbal command “Look!”	2. Attention: **form**, gaze to image of joint attention
3. Prompt performance: **form**, “What’s that?”	4. Act: **form**, label, “Fishy”
5. Binary feedback + prompt performance: **form**, positive feedback and label, “Yes, and what’s he doing?”	6. Act: **form**, words
7. Binary feedback: **form**, “Yes”	

*The parent decides if and how often steps (4) and (5) of Frame 1A occur. The parent decides how often steps (3) and (4) of Frame 1B are repeated. The parent decides how often steps (5) and (6) of Frame 1C are repeated*.

#### 2.3.1 Bruner (1983)

##### 2.3.1.1 Focus

Bruner observed a mother and her son during the natural occurrence of (picture) book reading during the child’s second year of life.

#### 2.3.1.2 Interaction Category

The interaction is about explicit training and can be lead by both the parent and the child. The parent directs the child’s attention, but the child can also initiate the interaction by first pointing to an image, for example. The parent guides the infant to produce an acceptable label.

#### 2.3.1.3 Pragmatic Frame

For the book-reading pragmatic frame, Bruner (1983) (p. 78 ff.) identified a set of acceptable tokens for the mother’s utterances (Table 6). Similar to our analysis, he also classifies her utterances into key utterance types, which we will here present with their most frequent tokens (≥10%) (taken from Bruner (1983) (p. 78 ff.)):

- *Attentional Vocatives*, “Look!” (94%);
- *Queries*, “What’s that?” (67%);
- *Labels*, “X (=a stressed label, a noun for a whole object)” (42%), “It’s an X” (16%), “That’s an X” (13%);
- *Feedback*, “Yes” (63%), “Yes, I know” (10%).

The most flexibility of form is thus present in the presentation of the label (i.e., the learning content) and thus in the slot. Positive feedback is the common type, and negative feedback with or without correction is much less frequently given (only 15% of feedback utterances). The child participates with vocalization, gesture, smile, eye contact, and search for object. Not surprisingly, his participation increases with age and changes as, for example, undifferentiated deictics develop to pointing gestures. The initialization of the interaction can also come from the child who points to an image resulting in the omission of the attentional vocative the mother usually initiates the interaction with. The mother determines how often she will ask for the label, as she concludes the interaction when she is satisfied with her son’s performance. Importantly, the mother’s and child’s turns appear in a sequence and only overlap by accident (about 1% of the time).

Concerning the development of the pragmatic frame, Bruner (1983) (p. 124) describes that the mother adjusts the level of variability and difficulty to her son’s age and capabilities: “The mother restricts the task to the degrees of freedom that she believes the child can handle, and once he shows signs of doing better than that, she raises the level both of her expectancies and of her demands on the child.”

At first (Table 6, Frame 1A), the child only produces babble strings and smiles for his turn upon which the mother utters positive feedback and the correct label.

Then (Table 6, Frame 1B), with the appearance of standard lexical labels, she is only satisfied with the child’s answer, when he produces a lexeme-length babble. As soon as her son acquired the capability to produce words, she instead insists on words.

Another big change happens, when the mother knows that the child knows the label she asks him for (Table 6, Frame 1C). Then, her intonation pattern in the query (“What’s that?”) switches from a rising to a falling intonation. The child then gazes at the mother, smiles, and to tease her delays his answer a bit. With her positive feedback, she would then elaborate comments and questions for new information (e.g., “What’s that?” “Fishy.” “Yes, and what’s he doing?”; the rising intonation which was previously on the labeling query now shifts to “doing” in the new turn), developing the frame from labeling to predication.

#### 2.3.1.4 Implicit Knowledge

There is no implicit knowledge. The pragmatic frame itself however carries important information for learning (where the learning content is, what type it is, and how to process it).

Table 7 summarizes shared points and highlights the major shortcomings of the various presented papers. Compared to natural adult–child interaction (final row of the table) like the one we describe above, only few rigid frames are used in the robot learning approaches which in general are not learned.

**Table 7:** **Summary table of major commonalities and limits of the discussed literature**.

Work	Number of frames	Number of information types (user and robot)	Number of communicative cognitive functions	Degree of flexibility of frame	Number of elements of a frame learnt	Number of new frames learnt
Lallée et al. (2010)	1	8	2	None	0	0
Saunders et al. (2006)	2	9	2	None	0	0
Nicolescu and Mataric (2005)	2	7	3	Medium	0	0
Thomaz and Cakmak (2009)	1	5	1	Low	0	0
Yamashita and Tani (2008)	2	2	2	None	0	0
Calinon et al. (2010)	2	2	2	None	0	0
Mühlig et al. (2012) and Gienger et al. (2010)	1	10	2	None	0	0
Akgun et al. (2012)	1	8	3	Medium	0	0
Grollman and Billard (2011)	1	2		None	0	0
Lopes et al. (2007)	2	2	2	None	0	0
Kaplan et al. (2002)	2	8	2	Medium	0	0
Grizou et al. (2013) and Grizou et al. (2014)	1	2	1	Low	1	0
Steels and Kaplan (2002)	3	10	3	Low	0	0
Calinon and Billard (2006)	2	10	2	Low	0	0
Cakmak and Thomaz (2012)	1	8	1	Low	0	0
Natural social adult–child interaction, (e.g., Bruner (1983))	Many	Many	Many	High	All	All

## 3 Perspectives and Challenges for Future Research

Despite tremendous research efforts and advances in human–robot interaction with learning and teaching mechanisms, learning interactions with robots remain brittle and often highly pre-structured. There are many challenges to be addressed to allow for more natural and flexible interaction. Those which are currently gathering significant research efforts include the development of robot perceptual skills that allow robust multimodal recognition and tracking of social cues (Vinciarelli et al., 2012), rich verbal and non-verbal behavioral expression to convey understandable and usable information to non-expert humans (Lütkebohle et al., 2010; Lohse and van Welbergen, 2012; Salem et al., 2012), powerful statistical learning mechanisms that can generalize from limited input and identify patterns across modalities and time scales (Cuayáhuitl et al., 2015), adequate cognitive biases that can guide such inferences with, for example, intuitive physics and intuitive psychology (Lake et al., 2016), and even more basically physical bodies that are adapted to compliant and safe interaction with objects and people (Zinn et al., 2004; Sandini et al., 2007; Tsagarakis et al., 2011).

In this article, we have highlighted and discussed another fundamental challenge which has so far received less attention: understanding how robots could handle and learn through a rich system of pragmatic frames, constituting a grammar of social interaction for learning and teaching that may be as important as the concept of grammar for the use and learning of natural language.

We have shown that in many current approaches, some forms of pragmatic frames are used. However, such use has often been implicit, obfuscating the understanding of important dimensions of the mechanisms at work. Also, and more crucially, existing work has most often used only very few pragmatic frames in the same experiment (often just one), leading to rigid sequences of interaction, and it has used little potential cues conveyed by frames to bias the inference of learning algorithms. Hence, this has limited (a) learning capabilities and (b) interaction.

On the contrary, social learning in adult–child interaction has been shown to rely on the adaptive use of an open rich repertoire of flexible frames, facilitating learning by providing familiar contexts in form of coordinated action patterns that guide the children to pick up new action of language elements (Bruner, 1983). Furthermore, pragmatic frames in adult–child interaction constitute an evolving system where new frames are continually learned through mutual negotiation and alignment with caretakers, building cumulatively on interaction skills learned previously.

For robotics, such a rich and adaptive use of pragmatic frames promises to yield the following advantages: (a) a robot learner could benefit from the rich contextual information provided through the pragmatic frame for learning (Which information is important? What does it mean?); (b) a robot that could handle multiple pragmatic frames and even negotiate and learn new ones would allow for more natural interaction and flexible learning, being thereby more easily usable for inexperienced users.

However, there are many technical challenges to address in order to implement successfully such flexible mechanism in HRI with learning and teaching. In the following, we specify three challenges, each bearing a different aspect relevant to our perspective on flexible learning and teaching.

### 3.1 Handling Multiple Predefined Pragmatic Frames

The first challenge is how to enable robots to use multiple pragmatic frames, even if they might be hand-coded by engineers at the beginning (and in this case an open question is: which set of predefined frames shall be designed for a robot?). Using multiple of these frames in interaction with the human could allow the robot to dynamically switch between frames but entails solving several sub-challenges: The robot needs to detect which pragmatic frames are used by the interaction partner and to handle switches from the currently used frame. In addition, the robot needs to be able to propose a frame to its interaction partner, such that the frame is accepted and adopted, and to actively switch into a different frame. In order to achieve such a challenge, adequate repertoires of behaviors and perceptual modules need to be in the interactional toolkit of the robot so that it can flexibly use these frames in interaction and repair interaction when, for example, the human switches to another one.

A central scientific challenge is to understand what kind of computational representations shall be used and manipulated by robots, and covering both the syntax of pragmatic frames and their meaning by mapping elements/parameters of the interactional structure to adequate biases that will inform the inference process of learning algorithms. Examples of formal frameworks that could serve as a basis for these representations include the PaMini Framework (Peltason and Wrede, 2010), which consists of interaction patterns which share some aspects that are crucial for pragmatic frames (i.e., specification of recurring patterns, detection of patterns, interface to robot back-end) or the formal frameworks for construction grammar, which have already been suggested for extension to social interaction in general (Dominey, 2005, 2007).

Another central scientific challenge is how to design a system that can handle such a complexity and yet remain usable and efficient in its interaction with non-expert users. For example, techniques that would allow robots to initiate new pragmatic frames should be evaluated from the user’s perspective: how can it be correctly understood by the human and at what time during the interaction the initiation of a frame is appropriate and tolerated? If the users do not understand the frame as intended, do they ascribe a different frame to the situation? Or if they do not understand at all, how do they react?

Finally, the pragmatic frames considered are interaction protocols used to allow a robot to acquire target sensorimotor and language skills through interaction with a human. Even when pragmatic frames are already known, a respective architecture would entail using low-level learning mechanisms to acquire these target skills that can be adequately parameterized to benefit from the information contained in the interactional structure to bias their statistical inference (e.g., algorithms for learning motor skills should be able to get information about what aspects of the demonstrated behavior are important based on the interactional cues). For learning sensorimotor skills, Gaussian Mixture Models (or similar probabilistic models) could be used as a method to acquire new target motor skills, such as in state-of-the art methods for robot learning by demonstration [both for motor skills (Calinon and Billard, 2007) and language skills (Cederborg and Oudeyer, 2013)]. To acquire the meaning of new words, Bayesian inference techniques such as those presented in Xu and Tenenbaum (2007) could be used.

### 3.2 Strategically Choosing Which Frame to Use

A related challenge lies in giving the robot and human teacher the means to actively choose the most efficient pragmatic frame in the process of learning and teaching a new skill. Indeed, frames may carry different forms of information: e.g., one frame (possibly characterized by specific gestures or keywords) might cue that the teacher is trying to teach a new movement and providing information about its manner, and another frame could provide information about its goal or the conditions in which it shall be executed or not. In this context, a challenge is to let a robot know and learn which interaction frames should be used to teach/learn which skills. Thus, to approach this challenge, algorithms need be developed that allow the robot to estimate at each learning episode which target skill and which pragmatic frame should be used so as to maximize information gathering (and thus minimize the number of interactions needed to acquire the repertoire of target skills). Such estimations should be key for the robot either to decide to engage in an adequately chosen frame or to provide information to the teacher about its internal learning state so that the teacher can understand how to personalize/tune his/her teaching strategy.

As different skills and different frames are differentially difficult or useful to learn, random choices are bound to be highly inefficient. One way to address this issue is to formulate the problem of active selection of target skills and frames as an active learning problem, within the formal framework of strategic learning (Lopes and Oudeyer, 2012) developed in recent years in developmental robotics for learning motor skills in high dimensions (Baranes and Oudeyer, 2013), and more recently used in Intelligent Tutoring Systems to personalize teaching sequences (Clement et al., 2014). Such systems have been relying on the use of multi-armed bandits, which are used to make adaptive and active choices of the best current learning strategy (Nguyen and Oudeyer, 2012). As these techniques will allow to express various strategies both for the learner and teacher, human–human experiments need to be conducted and used to select those strategies that are closest to the ones spontaneously used by humans.

### 3.3 Learning an Unfamiliar Pragmatic Frame

Robots should also be prepared to be confronted with new frames which are not already in their repertoires. Two sub-challenges have to be differentiated here: (i) how to recognize a new frame against the background of familiar frames and (ii) how to learn the syntax and the meaning of a new frame. To develop a learning mechanism for learning the syntax of a pragmatic frame and to provide the back-end learning mechanism with information about the relevant slots that are given within this frame, the important steps are to allow a robot to detect that the human is using a new interaction structure, and learn the patterns of gestures, gazes, movements, and sounds, which rule its organization (its syntax).

For learning the meaning of pragmatic frames in the context of the acquisition of sensorimotor and language skills, an algorithmic framework should allow a robot to infer what kind of information is provided by a pragmatic frame. The first challenge is to develop adequate representations of the space of frame meanings so that it can be used operationally to bias the inference of a statistical learning algorithm used to learn a target skill or a target word. A possibility could be to use a Bayesian framework, where at the low-level, Gaussian Mixture Models (or similar probabilistic models) can be used as method to acquire new target motor skills or word meaning, such as in state-of-the art methods for robot learning by demonstration (both for motor skills (Calinon and Billard, 2007) and language skills (Cederborg and Oudeyer, 2013)). Such methods could allow to encode the meaning of frames as Bayesian priors over the space of motor skills or new words, and multiplicative operations over these priors are naturally capable of encoding the combination of multiple priors (such as, for example, when the meaning of a frame encodes an information such as “the target concept is a movement of the hand and the demonstration shows the goal”).

While one may consider incremental mechanisms which first learn the syntax of new frames, and then acquire its meaning afterward, an interesting target is to enable a robot to jointly learn the target concepts (e.g., a new movement or the meaning of a new word) and the meaning of a frame used to teach this concept (e.g., that this frame provides a particular information bias). This considers the problem where initially the learning robot will know neither the target concepts nor the frame meaning (so the robot will have no bias initially and face a large space of hypotheses for inference). A possible approach to this challenge relies on the use of expectation–maximization methods as those developed in Grizou et al. (2013) to allow a robot to jointly learn new skills and to interpret the meaning of new teaching signals.

### 3.4 Advancing the Understanding of Pragmatic Frames in Human–Human Interaction

While we have discussed here the question of how to transfer the concept of pragmatic frames to robot learning in interaction with a human teacher, there are many things we do not know about pragmatic frames in human–human interaction. More specifically, we know only little about how children react to novel pragmatic frames and how parents introduce them.

The load that a new situation brings about can be manifested by children’s inhibited behavior (Matthews et al., 2010; Beisert et al., 2012). Almost every word learning study is aware of the cognitive load trying to diminish it with a warm-up period preceding the experimental situation. Consequently, we know little about how children make sense of novel situations. Our own (Salas Poblete, 2011; Rohlfing et al., 2013) and others’ work (e.g., Moore et al. (2013)) provide first methodological approaches to actively manipulate pragmatic frames in the context of word learning and explore their influence on learning success. Clearly, the novelty of a situation imposes a greater cognitive load on children. It is likely however that children make use of the repertoire of frame they dispose of to understand new structures. This phenomenon becomes visible in adults (Vollmer et al., 2014). Thus, further adult–child experiments on interaction within new frames should shed light on this matter.

Also, experiments on human–robot interaction have the potential of contributing considerably to research on pragmatic frames in humans. Utilizing a fully controllable robotic system as a tool in the experimental design enables stable learner behavior and controlled manipulation of learner behavior for testing experimental conditions. Additionally, teachers in human–human interaction are never confronted with a learner that does not resort to an already acquired set of interaction protocols or only draws on a specific repertoire of pragmatic frames. Thus, it is interesting to understand how humans cope with such a robot learner and to investigate the emergence and negotiation of novel pragmatic frames in experiments with humans and robots.

## Author Contributions

A-LV, BW, KR, and P-YO developed the framework, reviewed the literature, and wrote the paper.

## Conflict of Interest Statement

The authors declare that the research was conducted in the absence of any commercial or financial relationships that could be construed as a potential conflict of interest.

## Footnotes

^1^In broad accordance with Clark (Clark and Brennan, 1991) by common ground, we mean the set of assumptions that the interaction partners share about the ongoing interaction. These assumptions may concern objects and actions as well as the interaction partner’s understanding of the situation and the communicative goals.

^2^As we only consider explicit learning/teaching interactions from the developer’s point of view (i.e., the developer/programmer/experimenter intended the interaction to be about learning and designed it accordingly), with this distinction, we refer to the teaching user’s intention. Explicit teaching hence relates to the user intending to teach the robot an action or word, whereas in implicit teaching, the user is not aware of the teaching interaction and, for instance, only provides the data for certain movements.
