# Supplementary material for: Pragmatic Frames for Teaching and Learning in Human–Robot Interaction: Review and Challenges
Source: Front Neurorobot. 2016 Oct 3;10:10. doi: 10.3389/fnbot.2016.00010 (PMC5046941; doi:10.3389/fnbot.2016.00010)
Supplement: Supplementary file 1 [file Presentation_1.PDF]

Supplementary Material: Pragmatic frames for teaching and learning in human-robot interaction: review and challenges

Anna-Lisa Vollmer *, **Britta Wrede, Katharina J. Rohlfing, and Pierre-Yves Oudeyer**
*Correspondence:
Anna-Lisa Vollmer:
anna-lisa.vollmer@inria.fr

1 SUPPLEMENTARY TABLES

The following tables depict the pragmatic frames for each analyzed work. The flexibility (see main text) of the information types or steps or elements of the frame are stated in bold. Information which has not been reported, which however is necessary for the interaction is stated in italics. Two steps of the frame with the same number occur somewhat simultaneously. 'Start' and 'end' are framing the interaction from the user perspective.

1.1 Passive learning - with symbolic representation of sequences of predefined actions

Table S1. Pragmatic frame for Lallée et al. (2010). The input always has the form of steps (10) to (13) or (6) to (9), in case the speech command has not been correctly understood, and continues for the sequence of actions the user teaches.

Experimenter/ Programmer actions	Teaching user actions	Robot learner actions	Robot learning
Frame 1			
		1. start: “I am ready”	
	2. input start: speech command “Learn.”	3. query: “You said learn?”	
	4. feedback: “yes”	5. confirm: “I’ll start learning now. I am ready”	
	6. input: speech command like “Prepare.”, “Left open.”,	7. query: “You said [command]?”	
	8. feedback: “No.”	9. confirm: “I am ready.”	
	10. input: speech command like “Prepare.”, “Left open.”,	11. query: “You said [command]?”	
	12. feedback: “Yes”	13. confirm and execute command: “Doing [command]. I am ready.”	
	14. input submit: speech command “OK”	15.query: “You said OK?”	
	16. feedback: “Yes”	17. confirm: “OK we will store this plan. I am ready.”	store executed sequence as macro
	18. prompt performance: “Macro”	19.query: “You said macro?”	
	20. feedback: “yes”	21. confirm and perform: “Running the macro. [command], [command] (execute commands) In line macro finished. I am ready.”	
22. end			

Table S2. Pragmatic frame for Saunders et al. (2006). Steps (3) to (5) can be repeated in order to build competencies hierarchically.

Experimenter/ Programmer actions	Teaching user actions	Robot learner actions	Robot learning
	Frame 1		
1. start	2. mode: button for training mode		
	3. input start: button ‘start learning’		
	4. input: prepare the environment and combine existing primitives from list to form a new action, provide label via keyboard		
	5. input end: button ‘stop learning’		store sequence
	6. prompt input performance, **optional**, **timing**: button for execution	7. perform input	
	Frame 2		
	1. mode + prompt performance, **optional**, **timing**: button for execution mode, select label for action from list	2. perform	
end			

Table S3. Pragmatic frame for Nicolescu and Mataric (2005). The experimenter decides how long each phase lasts.

Experimenter/ Programmer actions	Teaching user actions	Robot learner actions	Robot learning
Frame 1			
1. start			
2. phase	3. input start: speech command "start"		
	4. direct attention, **optional**, **timing**: speech command "here" + input, **timing**, **form**: lead the robot, speech commands "take" and "drop" to pick up and drop objects	4. execute commands: follow the user, pick up and drop objects	
	5. input end: speech command "end"		learn: store sequence
Frame 2			
1. phase + prompt performance		2. perform	
	3. Feedback, **timing**: Correct the performance, delete wrong elements in the sequence with the speech command "bad" and insert missing elements with the speech commands "come" to enter into and "go" to leave frame 1 for following and learning	3. execute commands	
end			

1.2 Passive learning - supervised

Table S4. Pragmatic frame for Thomaz and Cakmak (2009). Concerning the timing: The user decides how often which object is shown. How step (6) is communicated is not reported. The user either communicates directly with the robot or first communicates it to the experimenter who, in turn, communicates it to the robot.

Experimenter/ Programmer actions	Teaching user actions	Robot learner actions	Robot learning
	Frame 1		
1. start	2. direct attention+input, **form**: position one object centered in the workspace of the robot	3. input error: tilt neck to upper limit	
	4. direct attention+input: reposition the object centered in the workspace of the robot	5. act: preprogrammed motion	
	6. end		
7. input: hand-labeled affordances			train SVM classifiers

Table S5. Pragmatic frame for Yamashita and Tani (2008). There are five kinesthetic demonstrations ((3) - (17)).

Experimenter/ Programmer actions	Teaching user actions	Robot learner actions	Robot learning
Frame 1			
1. start			
2. mode			
3. start input	4. input: kinesthetic demonstrations		
5. end input			
steps (3) to (5) x4			learn neural network model
18. end			
Frame 2			
1. start			
2. mode + prompt performance		3. perform	
4. end			

1.3 Passive learning with prob. movement representation

Table S6. Pragmatic frame for Calinon et al. (2010).

Experimenter/ Programmer actions	Teaching user actions	Robot learner actions	Robot learning
Frame 1			
1. start			
2. phase			
3. start input	4. input: four kinesthetic demonstrations		trajectories learned with HMMs and GMR
5. end input			
6. end			
Frame 2			
1. start			
2. phase + prompt performance		3. perform: show action	
4. end			

Table S7. Pragmatic frame for Mühlig et al. (2012); Gienger et al. (2010). Step (4) continues until offset of step (5). Step (8) continues until the offset of step (9). The user presents the demonstrations three to eight times, but how this is incorporated into the pragmatic frame is not clear.

Experimenter/ Programmer actions	Teaching user actions	Robot learner actions	Robot learning
Frame 1			
1. start		2. attention: gaze at involved object(s)	
	3. direct attention: tapping on involved object(s)	4. attention: gaze at involved object(s)	
	5. input: demonstration like pick up an object and stack it on top of another	6. confirm: gaze at tutor	
	7. input submit: lift left hand	8. confirm: saliency based gazing	
	9. prompt performance: move objects to the side of the robot and lift both hands		learning (GMM)
		10. performance error: verbally announce an error in the simulation of the movement	
	11. prompt performance: raise left hand	12. perform: grasp object(s), imitate action, release object(s), step back, and gaze based on saliency	
13. end			

Table S8. Pragmatic frame for Akgun et al. (2012).

Experimenter/ Programmer actions	Teaching user actions	Robot learner actions	Robot learning
Frame 1			
1. start			
2. mode	3. start input: verbal command ”New demonstration”		
	4. input: moving the arms of the robot and recording poses as keyframe with the command “Record frame”		
	5. end input: verbal command “End of demonstration”		
	6. prompt performance, **timing**, **optional**: verbal command “Can you perform the skill?”	7. perform	
Frame 2			
1.mode	2. prompt (input) performance, **timing**: verbal commands “Next frame”, “Previous frame”	3. perform	
	4. corrective feedback/input, **timing**: verbal commands “Modify this frame”, “Add new frame”, “Delete this frame”		
	5. prompt input performance, **optional**: verbal command “Play current demonstration”	6. perform	
	7. input submit: verbal command “Record this demonstration”		
	8. end		

1.4 Exploration learning with initial user demonstration

Table S9. Pragmatic frame for Grollman and Billard (2011). Steps (5) are repeated until success.

Experimenter/ Programmer actions	Teaching user actions	Robot learner actions	Robot learning
Frame 1			
1. start			
2. start input			
	3. input: two predefined failed kinesthetic demonstrations		
4. end input			represent movement as GMM
		5. act: exploration, perform a generated trajectory	5. update model
6. end			

Table S10. Pragmatic frame for Lopes et al. (2007).

Experimenter/ Programmer actions	Teaching user actions	Robot learner actions	Robot learning
	Frame 1		
1. start			
2. start input			
	3. input: demonstrations of affordances for all possible states of state-space, separate objects of different size, shape, and color with different actions (drop boxes into container, tapp small balls off the table, touch top of large balls)		
4. end input			classify actions according to known object affordance-based world model, learn reward function (Bayesian inverse reinforcement learning) and compute optimal policy
6. end			
	Frame 2		
1. start			
2. mode + prompt performance		3. perform: carry out action corresponding to initial state	
4. end			

1.5 Exploration learning with user refinement

Table S11. Pragmatic frame for Kaplan et al. (2002). Prelude: steps (2) to (32) are performed more than 30 times. Frame 1 steps (2) and Frame 2 step (3) are repeated until the user is satisfied.

Experimenter/ Programmer actions	Teaching user actions	Robot learner actions	Robot learning
	Prelude		
1. start	2.-32. feedback1+feedback2, **form**: predefined primary reinforcer like scratching/patting the head of the robot, secondary reinforcer like the utterance “Good”	33. confirm: ‘happy’ signal wagging its tail	learn association between primary reinforcer and words/sounds
34. end			
	Frame 1		
	1.start: verbal command “try”	2. act: movements like walking	
	2. feedback2, **optional**		reinforce current movement
	3. feedback1+advance sequence		
	4. input, **form**: label for movement sequence	5. confirm: blinking its eyes	associate label with reinforced behavior
6. end			
	Frame 2		
1. start	2. prompt performance: label	3. perform (modified) sequence	
	4. feedback1: primary reinforcer		
5. end			

Table S12. Pragmatic frame for Grizou et al. (2013, 2014).

Experimenter/ Programmer actions	Teaching user actions	Robot learner actions	Robot learning
Frame 1			
1. start		2. act: move in a grid world or pick and place cubes	
	3. binary feedback, **form**: good or bad		learn task and feedback-to-meaning mapping
or			
1. start	2. input, **form**: guidance like left, right	3. act: move in a grid world or pick and place cubes	learn task and feedback-to-meaning mapping
4. end			

Table S13. Pragmatic frame for Steels and Kaplan (2002).

Experimenter/ Programmer actions	Teaching user actions	Robot learner actions	Robot learning
	Frame 1		
	1. start: verbal command “stand up”		
	2. direct attention: verbal command “look” and present object	3. attention: gaze at object	
	4. input: label	5. input query: “[label]?”	
	or<br>Frame 2		
	4. prompt performance: “What is it?”	5. perform: “[label]”	
	or<br>Frame 3		
	4. performance+performance feedback: “Is it [label]?”	5. binary feedback(+corrective feedback): “yes” or “no; [label]”	
	6. binary feedback(+corrective feedback): “yes” or “no; listen; [label]”		reinforcement learning of object-word relation
7. end			

subsectionActive learning

Table S14. Pragmatic frame for Calinon and Billard (2006). Step (5) occurs at least once. The user decides how often s/he demonstrates the gesture. How step (9) is communicated is not reported. The user either communicates directly with the robot or first communicates it to the experimenter who, in turn, communicates it to the robot.

Experimenter/ Programmer actions	Teaching user actions	Robot learner actions	Robot learning
Frame 1			
1. start			
2. phase		3. act: show a gesture like pointing to an object	
4. start input	5. input, **timing**: gesture, imitating the robot		
6. end input			train HMM with demonstration(s) to represent each gesture
7. end			
Frame 2			
1. start			
2. phase	3. direct attention + act: point at an object	4. attend: gaze at the object	
	5. prompt performance: turn-taking signal - gaze at robot	6. perform: point to object	
		7. prompt feedback: gaze at user	
	8. binary feedback (+ corrective feedback): nodding or shaking the head (point at object again)		
	9. end		

Table S15. Pragmatic frame for Cakmak and Thomaz (2012).

Experimenter/ Programmer actions	Teaching user actions	Robot learner actions	Robot learning
	Frame 1		
1. start			
2. task	3. start input: verbal command “New demonstration”	4. confirm: speech and head nod	
	5. input: kinesthetic trajectory demonstration	5. attention: gaze to object	
	6. end input: verbal command “End of demonstration”	7. confirm: speech and head nod	
	8. start input: verbal command “New demonstration”	9. confirm: speech and head nod	
	10. input: kinesthetic trajectory demonstration	10. attention: gaze to object	
	11. end input: verbal command “End of demonstration”	12. confirm: speech and head nod	
	13. prompt input query: verbal “Do you have any questions?”	14. input query: pre-scripted	
	15. answer input query, **form**: verbal		
16. advance sequence: button press			
17. end			

REFERENCES

Akgun, B., Cakmak, M., Jiang, K., and Thomaz, A. L. (2012). Keyframe-based learning from demonstration. *International Journal of Social Robotics* 4, 343–355

Cakmak, M. and Thomaz, A. L. (2012). Designing robot learners that ask good questions. In *Proceedings of the seventh annual ACM/IEEE international conference on Human-Robot Interaction* (ACM), 17–24

Calinon, S. and Billard, A. (2006). Teaching a humanoid robot to recognize and reproduce social cues. In *Robot and Human Interactive Communication, 2006. ROMAN 2006. The 15th IEEE International Symposium on* (IEEE), 346–351

Calinon, S., D'halluin, F., Sauser, E. L., Caldwell, D. G., and Billard, A. G. (2010). Learning and reproduction of gestures by imitation. *IEEE Robotics & Automation Magazine* 17, 44–54

Gienger, M., Mühlig, M., and Steil, J. J. (2010). Imitating object movement skills with robotsa task-level approach exploiting generalization and invariance. In *Intelligent Robots and Systems (IROS), 2010 IEEE/RSJ International Conference on* (IEEE), 1262–1269

Grizou, J., Iturrate, I., Montesano, L., Oudeyer, P.-Y., and Lopes, M. (2014). Interactive learning from unlabeled instructions. In *Proceedings of the Thirtieth Conference on Uncertainty in Artificial Intelligence (UAI)*. EPFL-CONF-205138

Grizou, J., Lopes, M., and Oudeyer, P.-Y. (2013). Robot learning simultaneously a task and how to interpret human instructions. In *Development and Learning and Epigenetic Robotics (ICDL), 2013 IEEE Third Joint International Conference on* (IEEE), 1–8

Grollman, D. H. and Billard, A. (2011). Donut as i do: Learning from failed demonstrations. In *Robotics and Automation (ICRA), 2011 IEEE International Conference on* (IEEE), 3804–3809

Kaplan, F., Oudeyer, P.-Y., Kubinyi, E., and Miklósi, A. (2002). Robotic clicker training. *Robotics and Autonomous Systems* 38, 197–206

Lallée, S., Yoshida, E., Mallet, A., Nori, F., Natale, L., Metta, G., et al. (2010). Human-robot cooperation based on interaction learning. In *From motor learning to interaction learning in robots* (Springer). 491–536

Lopes, M., Melo, F. S., and Montesano, L. (2007). Affordance-based imitation learning in robots. In *Intelligent Robots and Systems, 2007. IROS 2007. IEEE/RSJ International Conference on* (IEEE), 1015–1021

Mühlig, M., Gienger, M., and Steil, J. J. (2012). Interactive imitation learning of object movement skills. *Autonomous Robots* 32, 97–114

Nicolescu, M. and Mataric, M. J. (2005). Task learning through imitation and human-robot interaction. *Models and mechanisms of imitation and social learning in robots, humans and animals: behavioural, social and communicative dimensions*

Saunders, J., Nehaniv, C. L., and Dautenhahn, K. (2006). Teaching robots by moulding behavior and scaffolding the environment. In *Proceedings of the 1st ACM SIGCHI/SIGART conference on Human-robot interaction* (ACM), 118–125

Steels, L. and Kaplan, F. (2002). Aibos first words: The social learning of language and meaning. *Evolution of communication* 4, 3–32

Thomaz, A. L. and Cakmak, M. (2009). Learning about objects with human teachers. In *Proceedings of the 4th ACM/IEEE international conference on Human robot interaction* (ACM), 15–22

Yamashita, Y. and Tani, J. (2008). Emergence of functional hierarchy in a multiple timescale neural network model: a humanoid robot experiment. *PLoS computational biology* 4, e1000220
